# Aligning syntactic structure to the dynamics of verbal communication: A pipeline for annotating syntactic phrases onto speech acoustics

**DOI:** 10.3758/s13428-025-02747-7

**Published:** 2025-08-07

**Authors:** Cosimo Iaia, Alessandro Tavano

**Affiliations:** 1 Department of Psychology, Johann Wolfgang Goethe-Universität Frankfurt am Main, Theodor-W.-Adorno-Platz 6, Frankfurt am Main, 60623 Germany; 2 CoBIC, Cooperative Brain Imaging Center, Johann Wolfgang Goethe-Universität Frankfurt am Main, Frankfurt am Main, Germany; 3 Department of Cognitive Neuropsychology, Max Planck Institute for Empirical Aesthetics, Frankfurt am Main, Germany

**Keywords:** Syntax, Alignment, Language, Neural tracking, Annotation

## Abstract

To investigate how the human brain encodes the complex dynamics of natural languages, any viable and reproducible analysis pipeline must rely on either manual annotations or natural language processing (NLP) tools, which extract relevant physical (e.g., acoustic, gestural), and structure-building information from speech and language signals. However, annotating syntactic structure for a given natural language is arguably a harder task than annotating the onset and offset of speech units such as phonemes and syllables, as the latter can be identified by relying on the physically overt and temporally measurable properties of the signal, while syntactic units are generally covert and their chunking is model-driven. We describe and validate a pipeline that takes into account both physical and theoretical aspects of speech and language signals, and operates a theory-driven and explicit alignment between overt speech units and covert syntactic units.

## Introduction

Three major assumptions underpin oscillatory approaches to naturalistic language experiments in neuroscience (Giraud & Poeppel, 2012; Gross et al., 2014). First, speech and language information units are sequentially concatenated (Kaufeld et al., 2020). Second, the human brain simultaneously tracks speech and language units (Ding et al., 2016). Third, speech and language units segregate to separate frequency bands of neural activity (Keitel et al., 2018; Kaufeld et al., 2020). Taken together, these assumptions posit the existence of a homology between time and information scales, whereby the hierarchy of unit-specific information, from individual speech sounds to full utterances, is mapped onto a corresponding hierarchy of timescales (Keitel et al., 2018; Coopmans et al., 2022; Kaufeld et al., 2020). The homology hypothesis was first proposed by Giraud and Poeppel (2012) and Ghitza (2011), limited to the segmentation of phonemes and syllables, for which a sequential combination of smaller into larger units is theoretically grounded (see Gwilliams et al., 2018, on look-forward processes in phoneme combinations). It is easy to obtain reliable timestamps for phonemes and syllables, since the boundaries between units are detectable thanks to their physical properties.

### Theoretical background

A major roadblock for all theories of speech tracking based on neural oscillations is that speech and language units are in a one-to-many relationship among each other: for example, the personal pronoun “I” in English can be chunked as a phoneme, a syllable, a word, or syntactically as a Noun Phrase (NP) (Kazanina & Tavano, 2023). In fact, it fits all these labels at the same time. This problem is rarely discussed for duration-based models of phoneme and syllable tracking, but it exacerbates once the homology hypothesis is extended to higher-level units, and particularly to structure-building units such as syntactic phrases or sentences (Norman-Haignere et al., 2022; Kaufeld et al., 2020; Coopmans et al., 2024; Zhao et al., 2024; Rimmele et al., 2021; Meyer et al., 2020b, a). Structure-building processes are based on the fundamental operation of syntactic recursion (Everaert et al., 2015), which has no theoretical temporal bound: consequently, syntactic units have no duration limit. For example, the instances “We”,“The ones writing”, and “The ones writing about syntactic recursion” are all categorized as NPs, with equal syntactic status albeit different internal complexity. While it is well known that language comprehension is bounded by human memory (e.g., Lewis et al., 2006) and articulatory effort limits, the cognitive operation of recursion is *per se* temporally unlimited, making abstract syntactic categories effectively atemporal.

If syntactic categories cannot be defined based on temporal statistics, then any homologous mapping of syntactic structure onto a neural timescale, as provided by neural oscillations, is impossible (Kaufeld et al., 2020; Coopmans et al., 2022; Kazanina & Tavano, 2023). Attempts have been made to reproduce the segregation of syllabic information within the theta band by projecting prosodic phrase duration onto the delta band timescale using non-naturalistic speech stimuli (e.g., number lists, Rimmele et al., 2021), and aided by the only apparent connection between prosodic units and musical phrases (Doelling et al., 2014). Such attempts are marred by their limited generalizability to natural language understanding, but together with temporal approaches to syntax (Kaufeld et al., 2020; Coopmans et al., 2022) they motivated us to devise a theoretically explicit, procedurally transparent, and methodologically valid analysis pipeline for the alignment of syntactic structure notation to the dynamics of speech acoustics.

**Fig. 1:**
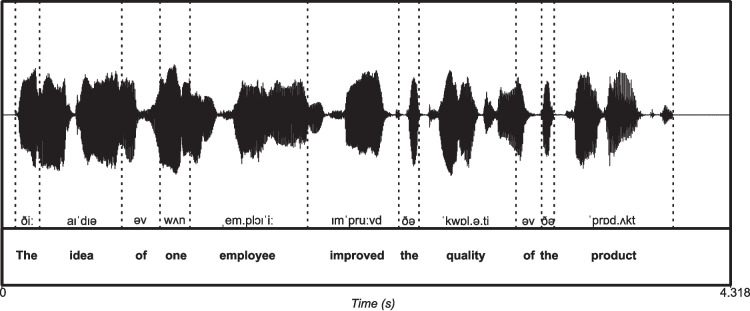
Word timestamps extraction. The acoustic signal is synthesized with gTTS (Durette, 2024) in Python, and then the annotation is performed manually in Praat (Boersma & Weenink, 2023). The temporal references of words are marked. Note that two words do not overlap on the time axis, i.e., their onsets and/or offsets never intersect, but follow one another sequentially

### Aim of the pipeline

Many available natural language processing (NLP) tools reliably extract phonemes, syllables, and words from the speech signal, and syntactic parsers offer a phrase structure analysis of the written text, but no NLP procedure helps with the alignment of syntax to the corresponding acoustic information. Furthermore, mining linguistic information for speech units and extracting syntactic phrases are fundamentally different processes. Given that speech units are not nested within each other (e.g., a phoneme is not nested within another phoneme), the temporal references of such units do not overlap on the time axis, thus making the extraction of their temporal references sequential. In contrast, syntactic units are instead hierarchical (or nested, see Everaert et al., 2015). Necessarily, the extraction of their temporal references implies a large degree of overlap of onsets and/or offsets of syntactic phrases at different nesting levels: e.g., in the phrase “The one writing this paper”, the offset of the word “paper” coincides with the ending of the NP “this paper”, the gerundial infinitive behaving as an NP “writing this paper”, and, of course, the NP “The one writing this paper”^1^. Importantly, temporal marking based on manual annotations of syntactic phrases is costly in terms of labor intensity and duration (a linguist specialized in syntax must be hired, e.g., Brennan et al., 2016). The majority of extant studies of naturalistic language tracking have predicated the extraction of structure-building units on arbitrary, or at least partially opaque, design choices, which negatively impact the reproducibility and computational accuracy of syntactic annotations. There is, at present, no consensus on how to mark the temporal boundaries of syntactic phrases on continuous speech. For example, in Keitel et al. (2018), (syntactic) phrase boundaries appear to roughly match the presence of prosodic pauses in the acoustic signal, thus making this process sequential, rather than hierarchical. In contrast, in Coopmans et al. (2022) the authors acknowledge the problematic move of sequentially marking syntactic phrases disregarding their nesting relationships, but do not directly address that crucial issue. We propose a pipeline to align parsed syntactic structures to their acoustic input in a systematic way. The pipeline extracts time-locked, sequential phrase annotations, while preserving the dimensionality of hierarchical structure. Finally, we provide the code and minimal working examples to apply this pipeline to other datasets.^2^.

## Timescales, dimensionality, and structure-building in speech and language

When we speak or listen to someone speaking, the building blocks of speech naturally unfold over time (see Box 1). With the exception of predictive processes at play during anticipatory coarticulation, which allow anticipating upcoming vowel targets before their acoustic onset (Farnetani & Recasens, 2010; Beddor et al., 2013), phonemic and syllabic combinatorial processes progress sequentially, unit by unit, on the time dimension. Although in natural speech the offset of the previous word does not always coincide with the onset of the next word, sequentiality in speech is guaranteed by the key axioms that a phoneme cannot contain another phoneme, and a syllable cannot contain another syllable (see Fig. 1).

On those premises, it is possible to retrieve the onset/offset markers of all speech units and compute their duration. Such information constitutes a set of duration distributions, which have been posited to constitute a hierarchy of timescales for the neural encoding of separate speech units (Gross et al., 2014). In particular, large-scale averaging analyses of the acoustic envelope have revealed a peak of energy between 3.5 and 5.4 Hz across several languages and attributed it to syllabic structure, suggesting that the human brain could exploit the implicit temporal regularity to track syllables in real time (Poeppel & Assaneo, 2020; Ding et al., 2017; Varnet et al., 2017). It is, however, unclear whether other units such as words also contribute to the acoustic envelope, as the issue has not yet been tested across several languages.

**Fig. 2:**
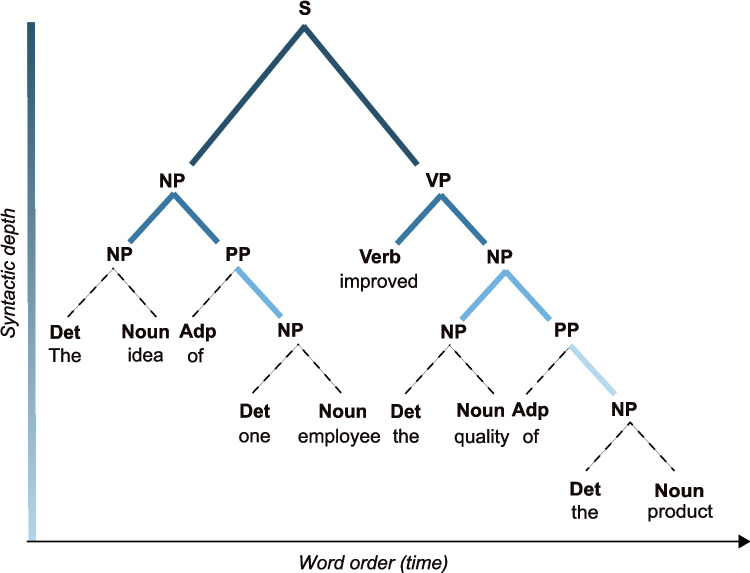
Visualization of a syntactic tree. Example of the syntactic tree for the sentence “The idea of one employee improved the quality of the product”. The labeling scheme is as follows: Sentence (S); Noun Phrase (NP); Verb Phrase (VP); Prepositional Phrase (PP). All other labels refer to the Part-of-Speech (PoS) of the words: Determiner (Det); Adposition (Adp); Noun (Nouns); Verb (Verb)

The main consequence of speech units’ sequentiality is that the combination of similar units will result in a higher unit, and thus a different scale. The combination of two phonemes results in a syllable but not in another phoneme. At the word level, however, a combination of two words can result in either a new word (e.g., a compound word) or a syntactic phrase (e.g., a Noun Phrase). The type of compositional operation that forms a compound word or a phrase is characterized by its degree of sequentiality: in the case of compounds, two words are typically combined into a new word that will act as a new unit of the same type (weak sequentiality), whereas in the case of a syntactic phrase (strong sequentiality), one item (the head) selects another item (a complement) resulting into a syntactic phrase (i.e., hierarchical structure, see Box 1). Hence, in the process of annotating the temporal references of words, given that both weak and strong sequentiality guarantee that there is no nesting of same-type units, word onset and offset will naturally follow each other, preventing overlap on the time axis (see Fig. 1).

## Projecting bidimensionality, preserving variability

Hierarchy in syntactic structure is a core assumption of many linguistic theories (Müller, 2023), among which phrase structure grammars (Chomsky, 1965; Everaert et al., 2015). In this type of grammar, in order to derive a full sentence, words have to be combined recursively, thus generating a hierarchical structure. As an example, take the following sentence:

1. labelitm:1 I like apples and detail its syntactic structure:
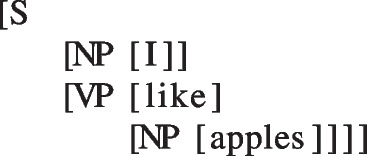
 it is clear that even for very simple sentences, phrases contain other phrases. Phrasal nesting becomes more apparent with complex sentences, such as the one depicted in Fig. 2:
3. The idea of one employee improved the quality of the product will result in the parsed structure:
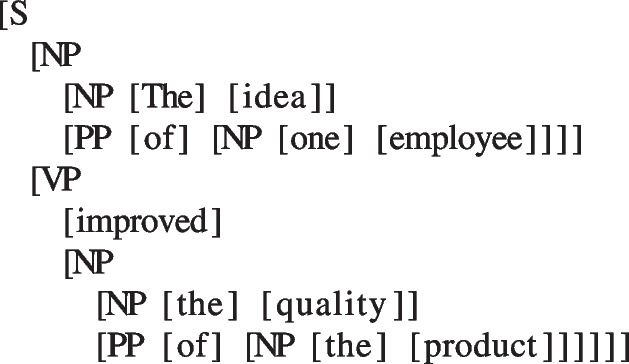

Examining the syntactic structure, it is apparent that the relationship among the items can be represented in a two-dimensional space in which the first dimension is word order, and the second dimension is the depth of the structure. The word-order dimension carries temporal information (i.e., word order on the time axis), whereas the depth axis maps the syntactic relations among all words and phrases in the structure.

**Fig. 3:**
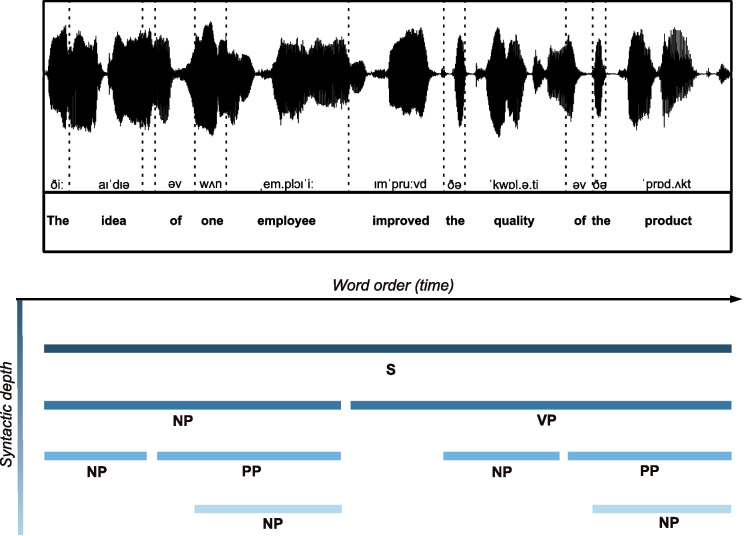
Alignment of word timestamps and syntactic structure. In this example, the higher-order phrases will share some of the onset/offset with lower-order phrases, as they are nested into each other. For example, the higher NP (the idea of one employee) has the same onset as the lower-left NP (the idea) and the same offset as the lower-right PP (of one employee) and NP (one employee). For this example, annotations at word level, as well as for the syntactic structure, were performed manually in Praat (Boersma & Weenink, 2023). The audio signal was synthesized using the gTTS (Durette, 2024) library in Python

Contrary to speech units (phonemes, syllables, and words), syntactic phrases can be combined with words or other phrases to form a phrase. That is, same-type units can be combined and, crucially, will derive a unit on the same linguistic scale of information. Consequently, the temporal references of syntactic phrases (both onsets and offsets) might overlap across different phrases on the time axis, as every phrase can be nested into another phrase (see Fig. 3).

When marking the temporal references of syntactic phrases, however, each node in the structure is superposed to one or more words (see Fig. 3). The NP “The idea” will have an onset and offset, so will the following phrases. In turn, this NP is contained within the larger NP “The idea of one employee” so that they will have the same onset but a later offset. Thus, the alignment between speech units and syntactic units is best seen as the alignment between the word timescale and syntactic structures. As syntactic structures are hierarchical, the bidimensionality resulting from the nesting process is projected onto the time axis, while preserving the variability of the onset/offset of these units.

## Design philosophy for the alignment of word timestamps with syntactic phrases

The Python pipeline we designed takes as input words’ timestamps (i.e., onset and/or offset) and a syntactic structure, using the bracket notation shown in Section “Projecting bidimensionality, preserving variability”, taken as a string rather than a parsed object. The rationale behind such a design choice is that not all NLP tools are available for all languages. For example, the Penn Phonetics Lab Forced Aligner is a very popular tool to mark word timestamps in the English language, as it automatically extracts them given the text transcription (Yuan & Liberman, 2008). However, the same tool is not available for all languages, other than English. In such a case, the temporal references need to be extracted in a different way, for example by using Google Speech-to-Text or another forced aligner (e.g., McAuliffe et al., 2017). Ultimately, in user-specific cases, manual annotations of word timestamps might be preferred over automatic techniques for the sake of accuracy.

The same logic applies to syntactic analyses. Syntactic parsers with trained models are available for many natural languages, but with a different degree of accuracy, and in different programming languages (e.g., Java, Python), and, most importantly, with different parsing strategies (see Box 2). Additionally, some manual corrections might be needed when checking the parsing output, or a custom syntactic analysis might be preferred to meet specific experimental goals (i.e., testing a specific version of a phrase structure grammar). It is important to highlight that, given that a parsed structure is provided as an input for the following pipeline, the mapping algorithm between word timestamps and syntactic phrases is independent from the parsing strategy (top-down, left-corner, bottom-up) and the specific library used, as long as the structures are in bracket notation (see Box 2).

**Fig. 4:**
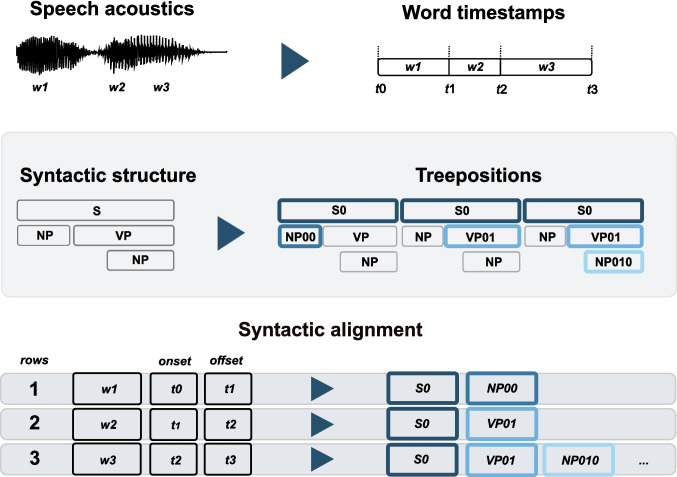
General overview of the pipeline. Given word timestamps and the parsed syntactic structure, the pipeline provides explicit alignment by tagging every word with all syntactic node labels necessary to integrate that word into the sentence. In order to uniquely identify each syntactic node label, its relative position (i.e., *treeposition*) is added. The final output is a dataframe that contains the word timestamps aligned with the syntactic structure, such that every word is tagged with all relevant information

### General overview of the pipeline

To align the word timescale and the syntactic phrase timescale, the core function we propose takes advantage of two popular libraries in Python, |stanza| (Qi et al., 2020) and |NLTK| (Bird et al., 2009). As a first step, a sentence is parsed into a syntactic tree making use of already implemented models in |stanza|. Note that |stanza|, as well as |NLTK|, can also leverage the Java implementation of CoreNLP that has more models available (Manning et al., 2014). Additionally, the |stanza| library has a built-in function for reading a string into a syntactic tree object |treereader|, which can be used to import parsed structures coming from other libraries or manual syntactic annotations, as long as the input structure is a string in the conventional bracket form. Therefore, the syntactic analysis used for the subsequent part of our pipeline does not depend on the specific library or a specific type of parser (top-down, bottom-up, or left-corner). For example, if a model for a given language is not available in |stanza|, the text material can be parsed using another library or can be manually annotated. The parsed tree can be converted into a string and imported in |stanza|, using the dedicated |treereader| function, to continue with the next steps of our analysis pipeline. Alternatively, syntactic analysis can be performed in Java or using the CoreNLP models, which are available in both Stanza and NLTK.

**Fig. 5:**
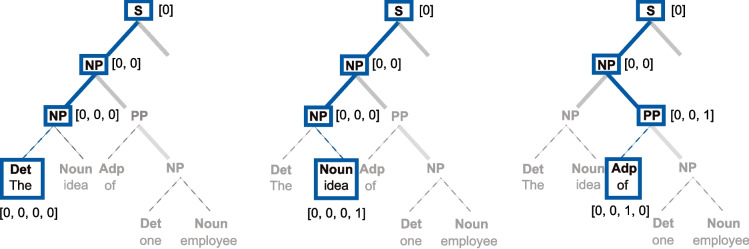
Example of using the ***treepositions*** to navigate a tree. For every word in a sentence, one loops through the structure and extracts the relative *treeposition* for every node in which the word appears. The indices of each label are used as a unique identifier for each node in the structure

### Step 1: extraction of the coordinates of leaves on trees

The basic idea behind the pipeline is that one can look at a syntactic tree as a series of nested lists that can be indexed. As one moves along a syntactic tree, each index becomes a coordinate for navigating the tree. First, the |stanza.tree| object is converted into a string and then read again into |NLTK| using the constructor |Tree.fromstring()| included in the Tree object. This step ensures compatibility with other parsers, as the parsed structures can be imported simply as strings. As a starting point, the method |Tree.treepositions(‘leaves’)| from the |NLTK| class will extract the exact coordinates of every terminal item in the syntactic tree. The output of this function is the index (or *treeposition*) of every word nested into the tree (see Fig. 5).

### Step 2: extraction of the label of each node in the hierarchy

Once the *treepositions* of the tree are extracted, we revert to the tree object in |stanza|. A tree object in |stanza| allows accessing every item in the nested structure by using the coordinates retrieved in Step 1. Simply put, the *treepositions* extracted in step one become coordinate indices to retrieve the labels of every item in the path to the terminal nodes (i.e., the words at the bottom of the structure). To extract every phrase label in the structure, we slice the *treeposition* relative to each word incrementally. For example, a word that has as *treeposition* = [0,0,1] will result in three relative indices ([0]; [0,0]; [0,0,1]), since every relative index corresponds to a node in the syntactic tree (see Fig. 5). To do so, |tree.children[index].label| stores the label of the current node while navigating the tree. To clearly distinguish each label by its relative *treeposition*, a relative index is added to each label, serving as a unique identifier.

### Step 3: Alignment between syntactic labels and word timestamps

Once all the nodes have been labelled and assigned with a unique identifier to each label, the result is a dataframe of objects for which every line corresponds to a word and every value to a unique label that is a combination of the syntactic node and its corresponding *treeposition* (see Table 1). Note that the length of the *treeposition* of the words (or terminal nodes) corresponds to the depth of the structure for that word. The final step simply aligns the syntactic words with the word timestamps (see Fig. 4).

**Table 1:** Syntactic annotations aligned at word level for the sentence example

onset	offset	words	0	1	2	3	4	5	6
0.079	0.223	The	S/0	NP/00	NP/000	DT/0000	The/00000		
0.223	0.710	idea	S/0	NP/00	NP/000	NN/0001	idea/00010		
0.710	0.936	of	S/0	NP/00	PP/001	IN/0010	of/00100		
0.936	1.114	one	S/0	NP/00	PP/001	NP/0011	CD/00110	one/001100	
1.114	1.810	employee	S/0	NP/00	PP/001	NP/0011	NN/00111	employee/001110	
1.810	2.351	improved	S/0	VP/01	VBD/010	improved/0100			
2.351	2.472	the	S/0	VP/01	NP/011	NP/0110	DT/01100	the/011000	
2.472	3.046	quality	S/0	VP/01	NP/011	NP/0110	NN/01101	quality/011010	
3.046	3.198	of	S/0	VP/01	NP/011	PP/0111	IN/01110	of/011100	
3.198	3.272	the	S/0	VP/01	NP/011	PP/0111	NP/01111	DT/011110	the/0111100
3.272	3.977	product	S/0	VP/01	NP/011	PP/0111	NP/01111	NN/011111	product/0111110

Each numbered column, conceptually, corresponds to one level of nesting in the syntactic tree, i.e., the depth of the structure. Each node label is split across multiple lines to match all the words related to the syntactic label. Additionally, each label has been assigned a relative *treeposition* in the structure, thus acting as a unique identifier for the label itself. The table matches the output in the dataframe

### Handling multi-word tokenization for alignment

The English language has many contracted forms that are used regularly. In the case of languages that have contracted forms, some tokenizers will split a word into multiple tokens. Consider now the sentence in English:

5. I hadn’t thought that there was too much you didn’t like eating, yesterday, at my parents’.

In such a case, there are two possible scenarios: (i) The contracted words are already split and have different timestamps (i.e., *had* and *n’t* have different timestamps); (ii) A blank space between words is kept as a guiding principle for the segmentation (e.g., *hadn’t* has only one onset and one offset). In scenario (i), the alignment can be carried out as described in the sections above. In scenario (ii), a tokenizer is likely to split a word into two different items (e.g., hadn’t $\rightarrow$ had / n’t). In example 5, words retrieved by the syntactic parser do not match the number of word timestamps. Therefore, it is important to decide how to align the syntactic annotations to the word timescale. Such a decision depends upon the objective of the analysis. If the aim is to compute the duration of phrases, for example, the alignment can be done by merging the split words. For example, *had / n’t*, annotated only for the unsplit word, can be merged back by skipping the extra line generated by the syntactic parser. This will result in the loss of the lexical category of the negation *not*, which, however, is irrelevant for the higher syntactic structure as the word timestamp for *hadn’t* already takes into account the duration of both items. The same strategy can be applied to other types of multi-word tokenization processes in natural languages other than English. More specifically, a list of contracted forms for English can be set to automatically exclude the lines of the table that result in a mismatch.^3^. It is worth pointing out, however, that this process still requires visual inspection because it largely depends on how the word timestamps are extracted (e.g., *had* and *n’t* can be marked separately or as one item in Praat) and on the actual parser used for the tokenization. Ultimately, some manual exclusions of specific items may be required, for example, in cases where incorrect tokenization results from the NLP model.

### Technical validation

In order to validate our approach, we compared syntactic phrasal annotations generated by the automatic pipeline, with manually annotated syntactic phrases extracted from an audio dataset in the Italian language. The audio files annotated are the first chapter of *Il tribunale delle anime* and the first chapter *Il gioco del suggeritore* in their audiobook format by Donato Carrisi^4^. In total, 18 min of audio signal were annotated at both word and syntactic phrase levels. We manually annotated word timestamps for a total of 2983 words. For manual syntactic annotations, a first syntactic parsing pass was run in |stanza|, and then the temporal references of each phrase were manually marked in Praat (Boersma & Weenink, 2023). The resulting syntactic analysis was visually inspected and corrected if needed (e.g., a phrase is incorrectly labelled by the parser). Upon correction, 3046 phrases were extracted.

For automatic syntactic annotations, syntactic phrases for the same dataset were extracted by running the pipeline described above, resulting in 3093 phrases. Note that the mismatch of the number of phrases is due to minimal corrections during the manual annotation process.

The mismatch in the number of phrases between automatic and manual annotations is explained by the fact that manual annotations were corrected for accuracy. Furthermore, the raw syntactic analysis for both manual and automatic annotations is virtually identical because it stems from the same parser. To match the number of syntactic phrases extracted automatically to those manually annotated, we randomly dropped 47 phrases from the automatic one.

**Fig. 6:**
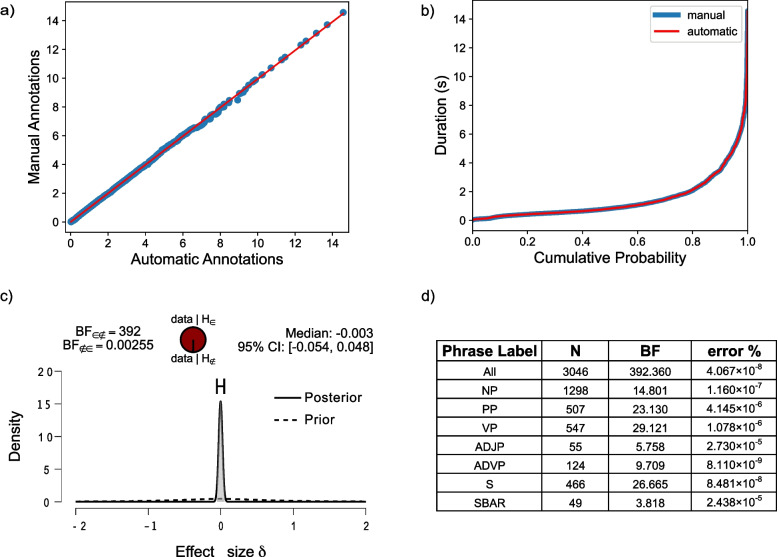
Comparison of durations of syntactic phrases extracted manually and automatically. **a** QQplot of the distributions of the durations of automatically extracted phrases and manually extracted phrases in seconds. **b** Empirical cumulative probability distribution of both automatic and manual annotations; **c** Equivalence prior and posterior for all phrases. **d** Equivalence Bayesian independent-samples *t* tests for all phrase labels. In case of mismatch in the number of items to compare, we resampled the data

Then, the duration distributions of manual and automatic phrases were compared by looking at quantiles of both distributions, assuming that the distribution of manually annotated phrases is the ground truth distribution (see Fig. 6). The Q-QPlot revealed an almost complete agreement between the two distributions. To further validate the pipeline, we ran an equivalence Bayesian independent-samples *t* test (in Jasp, JASP Team, 2024) at 95% credible interval (prior: Cauchy distribution, scale = 0.707). This resulted in a Bayes factor (BF) of 392.360, which is decisive evidence in favor of the hypothesis that the two sample distributions are statistically equivalent.

## Potential applications: Extracting frequency ranges from annotations for syntactic phrases

The main application of this pipeline is to correctly track the timing of structure-building operations in continuous speech, extending the scope of previous literature (e.g., Coopmans et al., 2022; Kaufeld et al., 2020; Keitel et al., 2018) to large-scale neuroscience experiments and NLP studies in a standardized and theoretically transparent way, eliminating the need for manual annotations. The temporal information related to main speech units (phonemes, syllables, words) is easily identifiable by sequentially marking their onset and offset: Once information about the duration of syntactic units in natural speech is computed, it is easy to convert it onto the frequency domain (F = 1/T), and frequency ranges can then be used to narrow-band filter the speech and the M/EEG signal, before calculating acoustic and neural envelopes, respectively. Such an approach is valuable to investigate the involvement of a particular timescale or, more interestingly, a combination of timescales in tracking language units. One can use the pipeline illustrated above to map every syntactic phrase to the word(s) and/or phrase(s) it contains, thereby easily implementing the analysis of recursivity, a fundamental but missing step in NLP pipelines. In the output table, all phrases are marked by their relative *treepositions*. This means that each node’s phrase duration will be equal to the sum of the duration of all words that are marked with the same node label at a specific degree of nesting (i.e., in each column of Table 1):

$$ Phrase Duration(node Label) = \sum _{Label(word) = nodeLabel} Duration(word_i) $$

In Table 1, the NP/00 duration would be equal to the sum of the duration of all words that have the same node label in column 1. Note that by computing phrase durations as the sum of each word duration, pauses between words will be filtered out. An alternative approach to retain pauses is to compute the duration of every phrase as the offset of the last word tagged with a phrase label minus the onset of the first word tagged with the same label.

$$ Phrase Duration(nodeLabel) = \begin{array}{c} \text {maxOffset(word)} \\ _{Label(word) = nodeLabel} \end{array} - \begin{array}{c} \text {minOnset(word)}\\ _{Label(word) = nodeLabel} \end{array} $$

In Table 1, the NP/00 duration would be equal to the offset of the word “employee” minus the onset of the word “The”. Note that the onset of “The” and the offset of “employee” correspond to the timestamps of the NP/00, making this approach suitable for extracting timestamps of all phrases without computing their duration. To show a potential application, we applied our pipeline to an already available dataset that has been used in previous studies, Alice in Wonderland (Brennan, 2023). The dataset already contains word onset/offset timestamps. Additionally, words are annotated in their split forms. For each sentence contained in the set, phrase information has been extracted by applying our pipeline. Phrase duration is then computed, retaining pauses within phrases, as described above. This resulted in a range of phrase durations between 0.048 and 22.89 s. This range of durations corresponds to a range between 0.044 and 20.97 Hz in the frequency domain.

## Discussion

We developed a pipeline to automatically mark the temporal references of syntactic phrases onto spoken speech, thereby making principled, reproducible, and scalable structure-building analysis available for all natural languages. We validated our approach by showing that the pipeline’s results are virtually identical to manual annotations, effectively superseding the major obstacle of labor-intensive human annotation time in naturalistic, large-scale, cross-linguistic studies.

Our pipeline solves two important issues in current approaches to continuous syntactic processing. The first issue concerns replicability and reproducibility. In previous studies (Coopmans et al., 2022; Kaufeld et al., 2020; Keitel et al., 2018), the phrasal timescale was annotated according to specific design choices, often opaque (not fully available) to the reader, and therefore not reproducible. This is a known and general problem with manual annotations, as they are tailored to the analysis of a specific stimulus set and/or a specific research question. A manual annotation process, besides being time-consuming, becomes particularly difficult to reverse-engineer when annotations are not published, and the language unit marking process is not explicitly described, and theoretically motivated. In recent years, replicability and reproducibility of experimental designs, methods, results, but also computational models, have become highly paramount (Miłkowski et al., 2018). For a computational approach to be replicable and reproducible, it is of the essence to mathematically formalize every step of its pipeline (McDougal et al., 2016; Guest & Martin, 2021). This also includes the process of retrieving onsets/offsets and the duration of each unit. In sum, our pipeline presents with the following advantages: 1) it ensures reproducible and transparent annotation schemes, allowing for a direct comparison among studies without relying on idiosyncratic annotation schemes; 2) it is considerably faster than manually annotating a speech stream, while yielding comparable results (see Section “Technical validation”). Future research can take advantage of this pipeline both for meta-analyses of previous studies, as it now becomes possible to re-align syntactic structures to the speech input in a consistent way across stimuli and languages, and for the generation of larger stimulus sets, as phrases can be automatically extracted without labor-intensive manual annotations. This approach is particularly useful when analyzing naturalistic datasets with several hours of recordings (e.g., Gwilliams et al., 2023; Armeni et al., 2022; Nastase et al., 2021). In this regard, an additional point of discussion concerns the errors systematically occurring from parsing models. As mentioned in Box 2, parsers have a quantified degree of accuracy, which depends on the language and the actual implementation of the model. This will result in a percentage of syntactic structures that are not parsed correctly. While we provide a pipeline to largely automate the extraction of phrases using NLP tools and perform the alignment to speech data, we still recommend visual inspection of the parsed structures. Indeed, any error coming from the parser, if not corrected, will be translated to the alignment process. Given the implementation of our pipeline, manual corrections to the raw syntactic analysis are possible, as long as the corrected structures are in the conventional bracket notation form (see Section “General overview of the pipeline”).

The second issue is theoretical. As illustrated, syntactic structures are bi-dimensional in their information layout, and yet, for most of our purposes we need to project them onto the time axis. In some studies, phrases are simply flattened onto a temporal sequence (e.g., Keitel et al., 2018; Kaufeld et al., 2020): Hence, the computation of plausible duration ranges cannot capture the nesting of phrasal units, and the information provided by the depth axis of hierarchical information is effectively discarded. Alternatively, one could count the number of closing nodes with every word in a sentence. The idea was first introduced in neuroscience by Brennan et al. (2012) and has been used in more recent work (Brennan et al., 2016; Coopmans et al., 2022). Furthermore, every word can be weighted with the depth of the tree structure, the number of opening nodes (Nelson et al., 2017), or the rate of the nodes (Coopmans et al., 2024). However, all these approaches, while retaining the information related to the incremental building of the structure, discard the temporal information of the mapped units (or add it as a separate vector, Coopmans et al., 2022). As a consequence, the specific timestamps of each phrase label are not easily retrievable. Our pipeline allows for the simultaneous retention of full-scale temporal information and hierarchical relations.

## Conclusion

Studying the dynamics of verbal communication requires correctly mining linguistic unit information. Recent advances in natural language processing (NLP) have increased the amount of linguistic information extracted from speech and text. However, the temporal alignment of higher-level units, such as syntactic phrases, to lower-level units – particularly spoken speech units – has proven elusive. We offer a pipeline to automatically and reliably align the temporal references of syntactic phrases (structure-building operations) and spoken words, a crucial step towards scaling a principled NLP analysis to larger datasets across many natural languages.

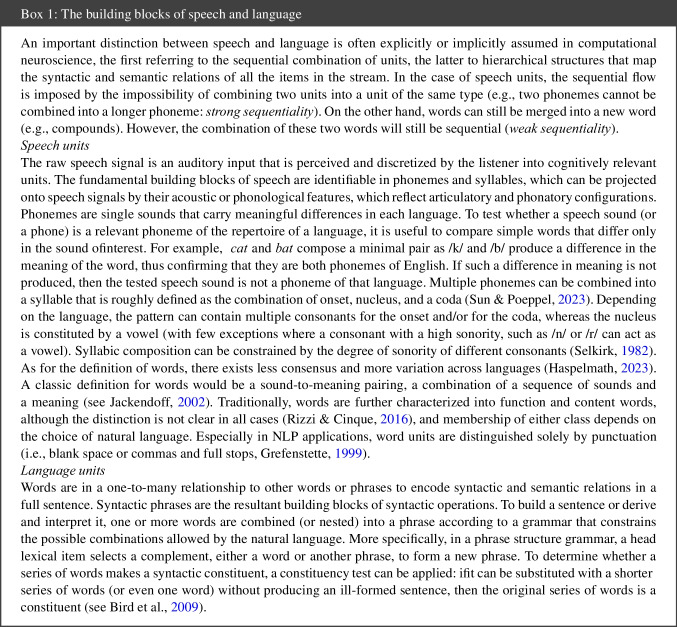

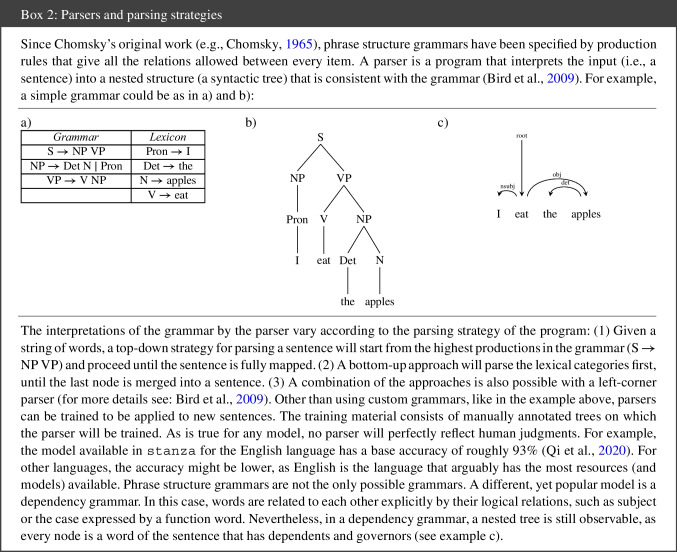





## Software

1. NLTK
2. stanza
3. pandas
4. Praat
5. gTTS
6. JASP

## Data Availability

Data for the manual annotations used for validation are available here: https://github.com/cosimo-iaia9305/align_syntax. Annotations for Alice in Wonderland are available here: https://deepblue.lib.umich.edu/data/concern/data_sets/bn999738r?locale=en.

## Footnotes

1 A long-standing debate in theoretical linguistics related to the proposal that the combination of a determiner and a noun results in a Determiner Phrase (DP) rather than a Noun Phrase (NP), where the lexical head selecting its complement is a determiner rather than a noun. This proposal was originally introduced to interpret Clauses and Nominal structures in a formally similar way, as the determiner would then operate akin to tense-marking elements for verbs (Abney, 1987; Bernstein, 2008; Longobardi, 2001).

2 The pipeline implementation is available here: https://github.com/cosimo-iaia9305/align_syntax

3 A minimal working example is provided here: https://github.com/cosimo-iaia9305/align_syntax/blob/main/Handling_contracted_forms.ipynb

4 Both audiobooks are read by Alberto Angrisano, and published by Salani Editore.

## Declarations

### Code availability

The code for aligning syntactic structures to word timestamps is available here: https://github.com/cosimo-iaia9305/align_syntax
